# Simulating individual movement in fish

**DOI:** 10.1038/s41598-023-40420-1

**Published:** 2023-09-04

**Authors:** Thomas W. Pike, Oliver H. P. Burman

**Affiliations:** Department of Life Sciences, University of Lincoln, Lincoln, LN6 7DL UK

**Keywords:** Behavioural ecology, Behavioural methods, Animal behaviour

## Abstract

Accurately quantifying an animal’s movement is crucial for developing a greater empirical and theoretical understanding of its behaviour, and for simulating biologically plausible movement patterns. However, we have a relatively poor understanding of how animals move at fine temporal scales and in three-dimensional environments. Here, we collected high temporal resolution data on the three-dimensional spatial positions of individual three-spined sticklebacks (*Gasterosteus aculeatus*), allowing us to derive statistics describing key geometric characteristics of their movement and to quantify the extent to which this varies between individuals. We then used these statistics to develop a simple model of fish movement and evaluated the biological plausibility of simulated movement paths using a Turing-type test, which quantified the association preferences of live fish towards animated conspecifics following either ‘real’ (i.e., based on empirical measurements) or simulated movements. Live fish showed no difference in their response to ‘real’ movement compared to movement simulated by the model, although significantly preferred modelled movement over putatively unnatural movement patterns. The model therefore has the potential to facilitate a greater understanding of the causes and consequences of individual variation in movement, as well as enabling the construction of agent-based models or real-time computer animations in which individual fish move in biologically feasible ways.

## Introduction

The way in which an animal moves is a fundamental component of its behaviour and ecology, underpinning how it interacts with both its biological and physical environments^1,2^. In fish, this has been most commonly investigated at the landscape level^3^ although finer-scale movements are known to play an important role in mediating short-term behavioural interactions. For example, behaviours such as predator inspection^4–6^, coordinated group movement^7^, and dynamic courtship displays^8–11^ require individuals to be acutely sensitive to how, where, and when other individuals are moving, and to adjust their own movement accordingly, often over small distances and short timescales. Indeed, fish have emerged as a particularly important group for understanding the link between fine-scale movement patterns and the evolution, function, and mechanisms underpinning social behaviours, and movement is (either implicitly or explicitly) an integral component of many empirical studies on fish social behaviour. For example, studies exploring the responses of live fish to robotic^12,13^ or computer animated^14–19^ stimulus fish typically recognise the importance of biologically plausible movement, and so design their stimuli to move in ways that elicit natural behavioural responses in observers. However, these movement patterns may be highly stylised (i.e., they utilise the general concepts of fish-like movement without considering the finer-scale components^12^) or based on a small number of exemplar movement paths which, while extracted from ‘real’ movement data^14^, may fail to encapsulate the variation inherent within the larger population or over time. They are also often designed to match how humans (rather than natural receivers, such as predators or conspecifics) perceive a given species’ movement ^16,20^.

Given the comparative paucity of appropriate movement data, we therefore have a poor understanding of the statistical rules underpinning individual fish movement at the spatial and temporal scales most relevant to behaviours such as shoaling, coordinated movement, and mate assessment (i.e., at a resolution in the order of millimetres and seconds, or finer)^21,22^. This not only potentially limits the comparisons that can be made both within and between species, but also precludes the inclusion of biologically plausible movement data in numerical simulations, and when controlling virtual or robotic stimulus fish.

The primary motivation of this study was to develop a biologically informed model of locomotory behaviour in three-spined sticklebacks (*Gasterosteus aculeatus*), an important model species in studies of behaviour and neuroethology^23^, which could be used to simulate their movement dynamics. Because fish intrinsically move within a three-dimensional environment and behavioural changes can occur very rapidly^24–26^ it was important to first quantify the three-dimensional movement of individual fish at a sufficiently high temporal resolution that movement paths could be created that encapsulate fine-scale movements. So that the resulting model could simulate a range of random, but biologically plausible, movement paths it was also important that the movement we quantified was representative of the variation present in the population. However, just because a model can recreate the statistical properties of data, it does not necessarily generate movement patterns that are perceived as ‘real’ ^27–29^. To address this, Herbert-Read and colleagues^29^ advocated the use of a Turing-type test in order to evaluate the effectiveness of animal movement models, by asking human observers whether or not they could distinguish between the movements of real animals and those simulated by a model. Here, we take this idea but pose it within a more biologically relevant framework, by presenting live sticklebacks with animated conspecifics endowed with either ‘real’ (i.e., empirically determined) or simulated movement patterns in a two-choice association task. To ‘pass’ the test we would expect live fish not only to respond to the simulated movement, but that their preference for simulated movement is statistically indistinguishable to that elicited by real-life movement.

## Results

### Characterising individual movement

**Figure 1:**
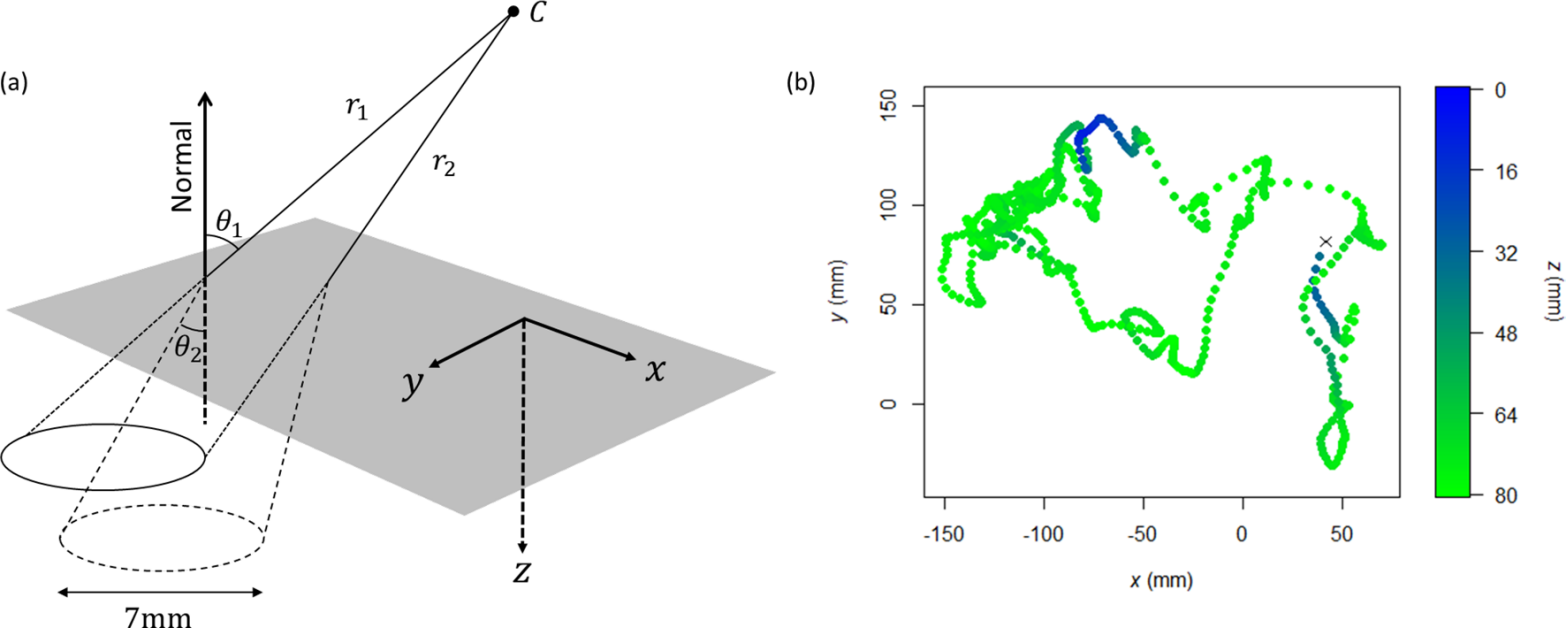
(**a**) Geometry of the tracking system, showing the true location of the circular white tag (dashed outline) along with its apparent location (solid outline) resulting from refraction at the air–water interface (denoted by the grey shaded region). $C$ denotes the virtual pinhole camera that produces the undistorted image (i.e., the hypothetical camera after removal of lens distortion), ${r}_{1}$ and ${r}_{2}$ are the two virtual rays cast from the camera’s centre of projection, and ${\theta }_{1}$ and ${\theta }_{2}$ represent the incident and refracted angles, respectively, of one of the rays. See text for full details. (**b**) Representative 60 s movement path segment. Each point denotes the fish’s position at 0.1 s intervals, with the colour representing depth ($z = 0$ is at the water’s surface). The fish started at the point marked ‘ × ’ and the centre of the tank was located at the origin.

**Figure 2:**
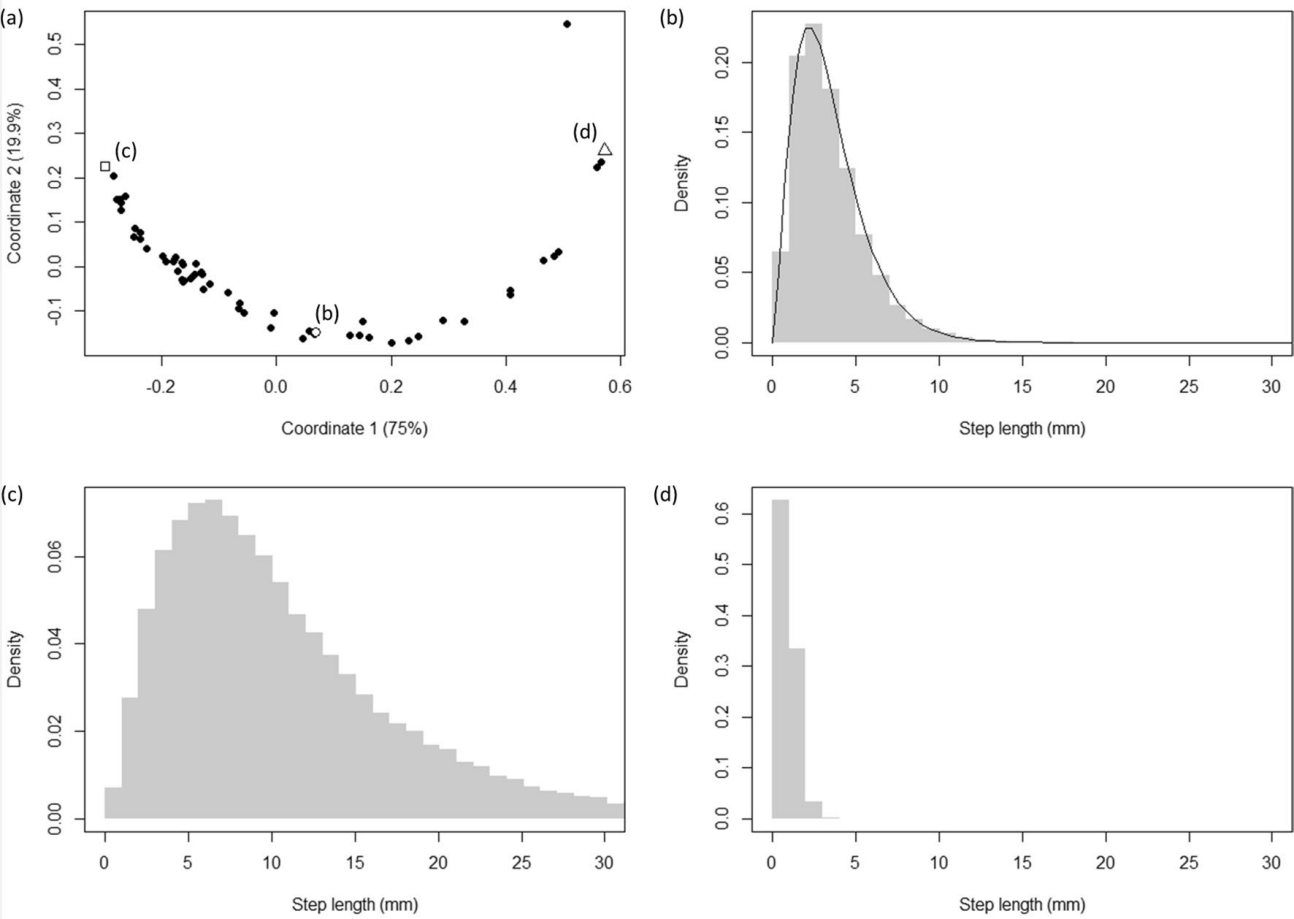
Summary of the observed inter-individual variation in step lengths. (**a**) Metric multidimensional scaling (MDS) plot showing the (dis)similarity between empirical distributions of step lengths. Each data point represents an individual fish, and histograms showing the distribution of step lengths over the 60 min period of data collection for the labelled data points are shown (**b**), (**c**) and (**d**). The histogram in (**b**) is overlaid with the best-fitting gamma distribution (solid line). The data point to the top right of the plot in (**a**) denotes a fish that exhibited almost no movement throughout the 60 min of data collection, and so is considered an outlier.

**Figure 3:**
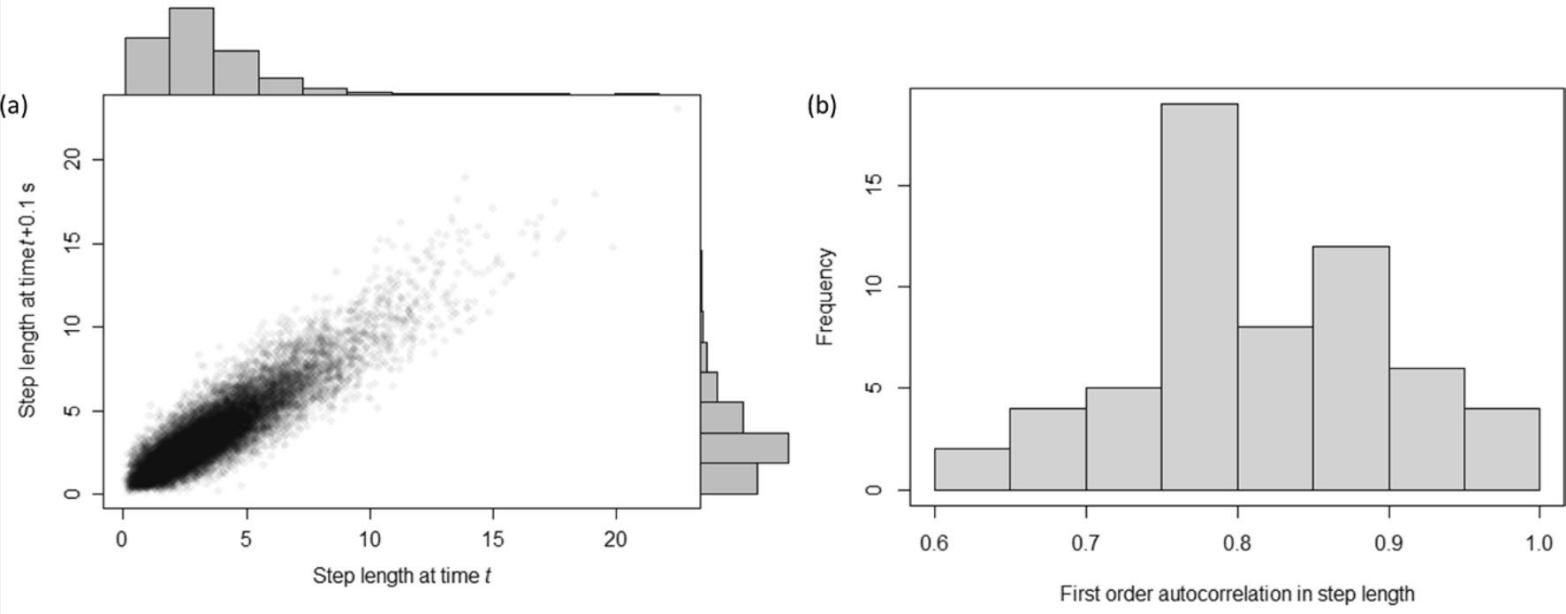
(**a**) First order autocorrelation of step length for a representative individual, with the histograms showing the marginal gamma-distributed steps lengths at each lag. (**b**) Distribution of autocorrelation values from the overall sample of 60 fish.

Individual fish moved in a characteristic saltatory manner, comprising forward movement in an approximately straight line followed by a stop, a brief pause and/or an abrupt change in direction (Fig. 1b). Although all individuals showed the same general pattern of movement, there was marked variation between individuals in how their step lengths and relative turn vectors were distributed (Figs. 2a, 3a).

In all cases step lengths could be well described by a gamma distribution (Fig. 2b), whereby the majority of steps tended to be relatively short (i.e., representing slow movement; clustered data points in Fig. 1b) occasionally interspersed with periods of longer steps (i.e., fast movement; spaced out data points in Fig. 1b). The median step length ranged between 0.20 and 9.15 mm (mean ± SD, 4.15 ± 2.05 mm) and the 95th percentile varied between 0.45 and 26.48 mm (mean ± SD, 11.45 ± 6.24 mm), indicating that some fish moved comparatively little (e.g., Fig. 2d), while others moved almost continuously for the entire data collection period (e.g., Fig. 2c). Fish also showed a very high degree of temporal autocorrelation in step length, with the mean ± SD of first order autocorrelations for the sample being 0.81 ± 0.08 (randomisation tests: all *p* < 0.001; Fig. 3).

**Figure 4:**
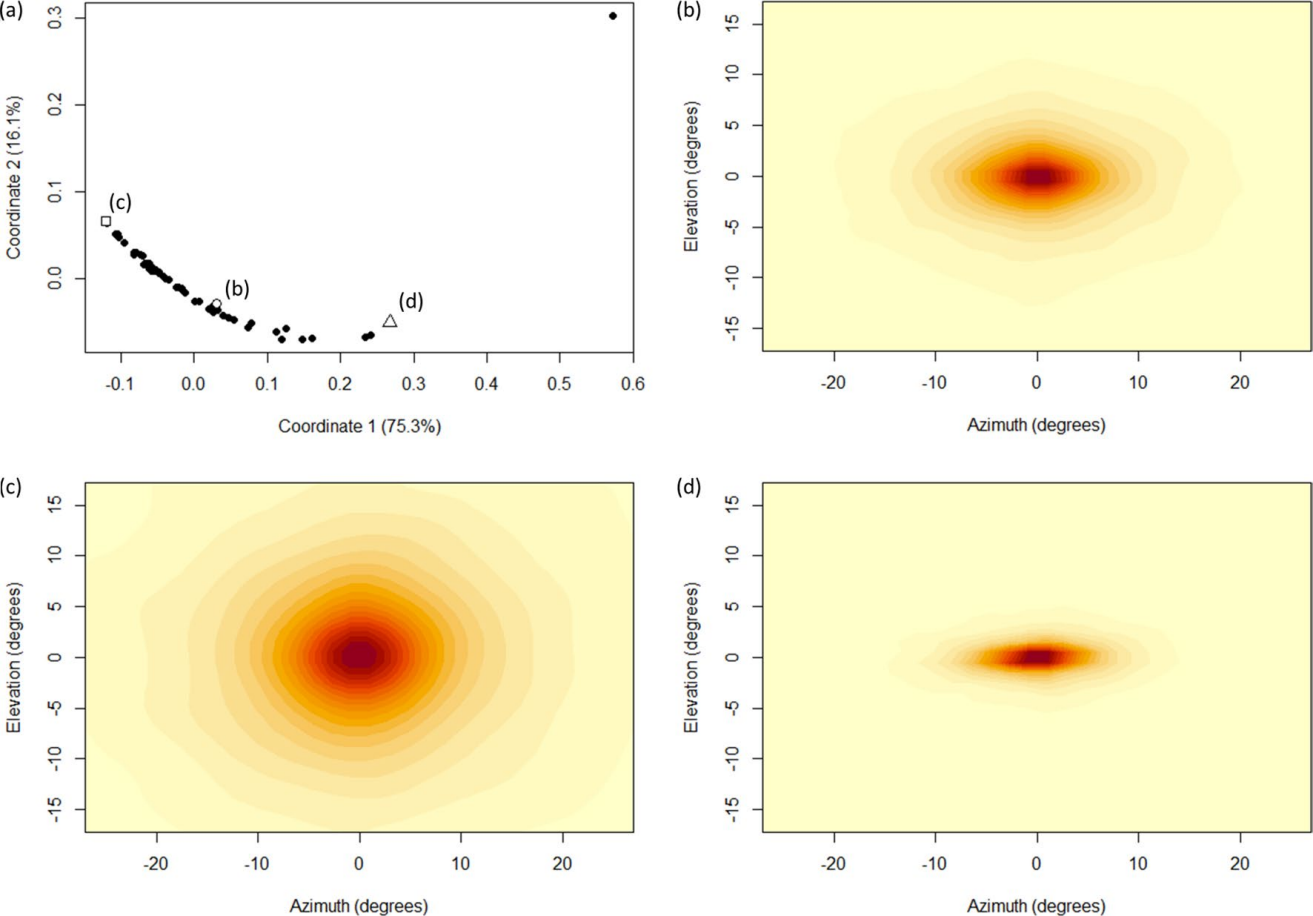
Summary of the observed inter-individual variation in relative turn vectors. (**a**) Metric multidimensional scaling (MDS) plot showing the (dis)similarity between empirical distributions of step lengths. Each data point represents an individual fish, with contour plots showing the distribution of relative turn vectors over the 60 min period of data collection for the labelled data points shown in (**b**), (**c**) and (**d**). In each case the spherical distribution has been ‘flattened out’ to show horizontal and vertical turns along the azimuth and elevation axes, respectively, and darker colours represent regions of higher density. The data point to the top right of the plot in (**a**) denotes a fish that exhibited almost no movement throughout the 60 min of data collection, and so is considered an outlier.

**Figure 5:**
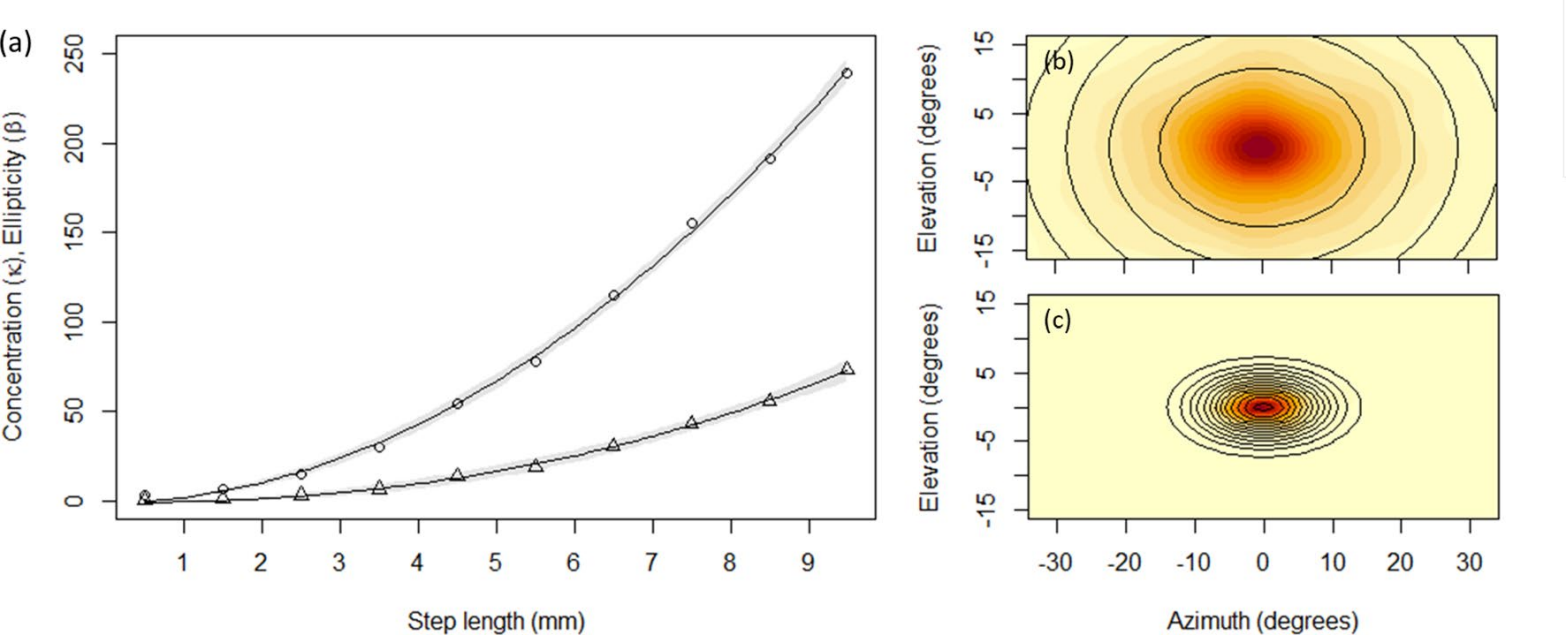
(**a**) Power relationship between step length (mm) and the concentration ($\kappa$; circles) and ellipticity ($\beta$; triangles) parameters of the best-fitting Kent distribution for each subset of the data, for a single representative fish. Data points denote the midpoint of each step length subset (breadth, 1 mm), with lines indicating the fit of the non-linear models and shaded regions the bootstrapped 95% confidence intervals. Only subsets containing ≥ 1000 data points were used. The interpretation of these parameters can be seen in the contour plots (**b**, **c**), which show the distribution of relative turn vectors over the 60 min period of data collection for step lengths (**b**) on the interval $[1 \mathrm{mm}, 2 \mathrm{mm})$ and (**c**) on the interval $[9 \mathrm{mm}, 10 \mathrm{mm})$, highlighting the increase in concentration (i.e., reduction in spread) and increase in ellipticity with longer step lengths. In each plot darker colours represent regions of higher density, and contours from the best-fitting Kent distribution have been superimposed (solid lines).

The distributions of relative turns tended to be elliptical, with greater spread laterally than dorsoventrally, and were distinctly leptokurtic, being characterised by a pronounced central peak indicating that fish predominantly maintained their current movement direction between successive time steps (i.e., the turn angle between successive time steps tended to be near zero, with only occasional turns at larger angles; Fig. 4b); although some individuals exhibited a much greater range and frequency of turns compared to others (e.g., Fig. 4c,d). Overall distributions of relative turn vectors were poorly described by single Kent distributions (Fig. 4b), primarily because the concentration and ellipticity parameters ($\kappa$ and $\beta$, respectively) were strongly predicted by step length (Fig. 5a). For all individuals, both concentration and ellipticity increased non-linearly as step lengths got longer, such that when fish were moving fast (i.e., had long step lengths) their movement was predominantly forwards with a comparatively small probability of making large turns. In contrast, when fish were moving relatively slowly, while their modal direction of movement was still forwards, they exhibited a much greater range of turn angles (Fig. 5b,c). The relationships between step length and both concentration and ellipticity were well described by power functions (Fig. 5a).

Body size (measured as standard length) did not predict the dissimilarity between distributions of either step length (distance matrix regression: pseudo-t = 0.005, *p* = 0.953) or relative turn vectors (pseudo-t = 0.036, *p* = 0.459), suggesting that the observed inter-individual variation in movement could not be attributed to differences in size between fish.

### Preference tests

**Figure 6:**
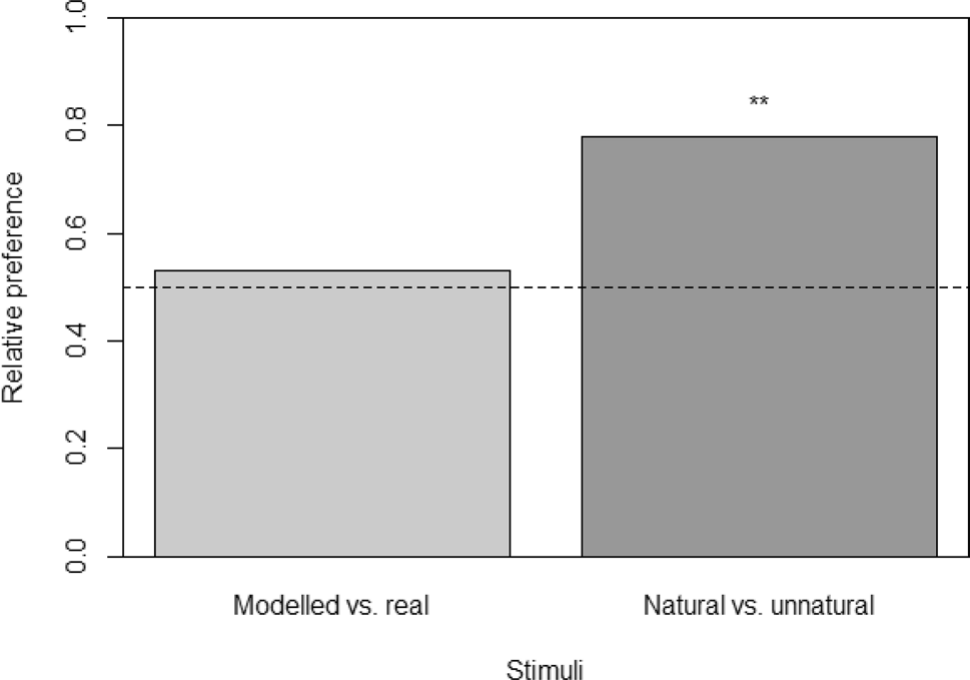
Relative proportion of live focal fish whose first-choice preference was for virtual fish displaying movement simulated using the model presented here, in trials where the choice was between modelled and real movement, and trials in which the choice was between modelled ‘natural’ movement and unnatural movement. Please see text for full details. Asterisks denote a significant difference from chance (i.e., a relative preference of 0.5), indicated by the dashed line: **, *p* < 0.01.

All focal fish exhibited a preference, by entering one of the choice zones within 90 s of the start of the trial (mean ± SD latency to respond, 11.07 ± 4.84 s), although the latency to enter a choice zone did not differ depending on whether that zone was in front of a virtual fish exhibiting modelled or real movement (modelled: 11.06 ± 4.23 s, real: 11.92 ± 4.71 s; two-sample *t*-test: *t*_30_ = 0.54, *p* = 0.591) or modelled versus unnatural movement (modelled: 11.15 ± 5.64 s, unnatural: 8.98 ± 3.52 s; *t*_30_ = 0.96, *p* = 0.345). Fish showed no significant first-choice preference, over and above chance, for virtual fish displaying movement simulated using the model over real movement (binomial test: *p* = 0.860), but significantly preferred virtual fish exhibiting modelled but ‘natural’ movement over unnatural movement (*p* = 0.002) (Fig. 6).

## Discussion

In this paper we quantify the three-dimensional movement of individual three-spined sticklebacks at a biologically relevant temporal frequency (10 Hz), using data collected from a representative number of individuals (n = 60) over comparatively large time spans (60 min). This allowed us to estimate key geometric characteristics of their observed movement in terms of the distribution of step lengths and relative turn vectors, as well as quantifying the strength of temporal autocorrelation in step length. The saltatory movement we observed is characteristic of three-spined sticklebacks^30^, and indeed animal movement in general^31^, and has been reported in a number of other fish species where it is typically associated with exploratory behaviour^32–34^. There was evidence of considerable between-individual variation in movement behaviour, with individual fish showing characteristic patterns of movement^35^ despite the consistency of the experimental context. Moreover, this encompassed much of the variation evident to human observers of stickleback behaviour (personal observation). While the underlying cause of this variation is not known, it is unlikely that it was caused by intrinsic factors such as the motivation to feed (not only did we attempt to standardise hunger levels prior to data collection, but previous work has found no difference in the locomotory behaviour of food-deprived and well-fed fish^36^) or extrinsic factors such as water temperature which, while known to affect activity levels in fish^37^, was held constant throughout the study. Furthermore, we could find no evidence that standard length predicted either step length or relative turn vectors, suggesting that the movement behaviour we quantified were not affected by body size (cf.^38^). Although not quantified explicitly here, there is evidence that the movement behaviour of individual fish is highly repeatable^35,39^, potentially indicative of personality differences, and there is growing evidence of individual consistency in (and correlation between) movement-related traits^40,41^.

The primary motivation of this study was to develop a biologically informed model of movement behaviour in fish, using three-spined sticklebacks as a model. The empirical data were used in the construction of a simple model of fish movement, which was capable of generating movement paths that we assume appeared biologically plausible to conspecifics. Certainly, when given a choice between two virtual fish, one following an empirically determined movement path and the other following a movement path generated by the model, they exhibited no significant preference either way. This is strengthened by the fact that live fish significantly preferred to associate with virtual fish exhibiting movement simulated by the model compared to fish that moved in what appeared to human observers to be an unnaturally stilted manner. This suggests that fish were not simply responding to the presence of a conspecific (in which case we would have expected their choice to be random) but could perceive differences in their movement and were choosing to associate with the fish that moved in the most biologically plausible way. We know that fish can make shoal choice decisions based on the behaviour of the stimulus fish (e.g., based on their overall activity levels^42^) and so it is entirely feasible that they could pick up on comparatively subtle differences in movement. Whether this was because they perceived it as ‘unnatural’ is not known, and it remains possible that they were in fact responding to perceived differences in movement indicative of a potential predation threat^43,44^, nutritional status^45,46^ or parasite infection^47^, for example. However, our results do highlight the potential utility of using Turing-type tests with animals to assess the perceived realism of simulated data, and this would be an interesting area to explore in greater depth.

To our knowledge there are no comparable, biologically informed three-dimensional movement models for fish at the temporal and spatial resolution considered in this study, and so this model has widespread potential for furthering both the theoretical and experimental understanding of fish behaviour. While it has been developed using empirical data for three-spined sticklebacks, it is likely to be applicable to a range of small fish species used in behavioural research, including zebrafish (*Danio* spp.), goldfish (*Carassius auratus*), and guppies (*Poecilia reticulata*). For example, it could be used to simulate biologically relevant movement in animated or robotic fish which, while widely used in studies on mate choice^14,15,17,19,48,49^, anti-predatory behaviour^50,51^, and shoal preference^18,52–54^, rarely try and emulate realistic movement patterns (although see^14^). Because the model can be used to generate movement in real time, it also has the potential to be reactive to the behaviour of the other (live or virtual) fish, and allow the creation of interactive experiments that incorporate feedback from biological or environmental events^15^. Furthermore, it could be used in spatially explicit individual-based movement models (e.g.,^55^), potentially increasing their predictive or explanatory power over more general approaches (such as correlated random walks^56^) and allowing for more realistic extrapolation over a range of spatiotemporal scales (from local up to landscape-levels^57^). Finally, it may be used to create null models in studies of animal movement, including the assessment of non-random grouping behaviour^58^. The model therefore has considerable potential to facilitate a greater understanding of the causes and consequences of individual variation in movement behaviour.

## Methods

### Source of fish and holding conditions

Adult three-spined sticklebacks were purchased from a commercial supplier (The Carp Co., Tonbridge, UK), and housed across several 50 L plastic tanks (at a density of approximately 0.6 fish L^−1^) filled with dechlorinated tap water. Each tank contained an air stone and sponge filter. The temperature of the room was maintained at a constant 8 ± 1°C, and the lighting schedule was a constant 12L:12D. The fish were fed to satiation daily on frozen bloodworm, and partial water changes were performed daily. They were housed under these conditions for approximately 6 months until the start of the study.

This study followed the ARRIVE guidelines^59^ and all methods adhered to the ASAB Guidelines for the Use of Animals in Research. The study was approved by the Research Ethics Committee of the University of Lincoln (reference CoSREC211).

### Collection of individual movement data

Data on the movement of individual fish were collected using the procedure described by AlZoubi et al.^60^. In brief, fish were individually marked by attaching a white circular tag (7 mm diameter and weighing approximately 8.4 mg) to the middle of their three dorsal spines^61^. This method of tagging does not affect behaviour^62^ and allows fish to be monitored under low light conditions against dark backgrounds^61^; conditions which are known to minimise stress in this species^63^.

Following tagging, individual fish were fed two large bloodworms (*Chironomus* sp. larvae) to standardise hunger levels, before being transferred to a black, circular test tank (30 cm diameter at the top, tapering to 27.5 cm at the base) containing 8 cm water^64^ and allowed to acclimatise for 60 min. Following acclimatisation, a remotely controlled overhead camera (GoPro Hero 3; GoPro Inc, San Mateo, CA) started recording video (1920 × 1080 pixel resolution, 30 fps) of their subsequent movements for a further 60 min. Lighting was provided by fluorescent ceiling lamps, located so that there was no glare from the water surface. In total, movement data was collected in this way from *n* = 60 fish (mean ± SD standard length, 41.8 ± 2.33 mm).

Custom tracking software was used to analyse the video footage and obtain information regarding the position of each fish at a sampling rate of 0.1 s, which corresponds to the response latency of fish^65^. Prior to the onset of data collection, we used the Matlab Camera Calibration Toolbox^66^ to estimate the intrinsic parameters (the effective focal length, principle point of the image plane, and lens distortion factors^67^) of the overhead camera by taking 20 images of a checkerboard pattern at different perspectives. This allows geometric distortion to be removed so that images can be treated as conventional pictures taken using a (virtual) perspective camera^68^. Furthermore, we also determined the extrinsic parameters of the camera (i.e., it’s rotation and translation) with respect to the surface of the water in the test tank, by imaging a checkerboard calibration target floating on the water’s surface^67^. Although we attempted to position the camera as close to perpendicular to the water surface as possible, this nonetheless allowed us to compensate for inevitable small deviations. The geometry of the tracking system is shown in Fig. 1a.

The tag was highly visible in each video frame, being white against the black background of the tank, and so could be located efficiently by first converting the frame to greyscale, and then isolating the tag using global thresholding with a pre-determined threshold^69^; the tag was always the largest fully connected region of pixels in the thresholded image. The edges of the tag were then identified using a Canny edge detector^70^, and the positions of the detected edge points corrected for distortion of the camera lens^71^. Because the perspective projection of the circular tag in any arbitrary orientation is an exact ellipse^72^, we next fitted an ellipse to the corrected edge coordinates using the least-squares approach described by Fitzgibbon et al.^73^, which provided information on the length and orientation of its semiminor and semimajor axes with subpixel accuracy^60^. This information could then be used to estimate the three-dimensional position of the tag (and hence the fish)^60,61^, as described below.

Regardless of the true orientation of the circular tag, the semimajor axis of the ellipse formed by its perspective projection in the image frame will always be directly proportional to the tag’s true diameter^74^, which is known precisely. It is therefore possible to find the three-dimensional position of the tag as follows. First, the two points on the periphery of the tag corresponding to the extremes of the semimajor axis are projected onto the water’s surface using the relationship

1 $$\mathbf{x} = \mathbf{X}\mathbf{R} + \mathbf{t},$$

where $\mathbf{x}$ is the (known) location of a point in image coordinates, $\mathbf{X}$ is the (unknown) location of the point in world coordinates, and $\mathbf{R}$ and $\mathbf{t}$ are the extrinsic parameters (see above) denoting the three-dimensional rotation and translation, respectively, of world coordinates relative to image coordinates^67^. Two virtual rays, cast from the camera’s centre of projection and each passing through one of the projected points, are then refracted at the air–water interface using Snell’s law, ${n}_{1}\mathrm{sin}{\theta }_{1}={n}_{2}\mathrm{sin}{\theta }_{2}$ (where ${\theta }_{1}$ is the incident angle, ${\theta }_{2}$ is the refracted angle, and ${n}_{1}$ and ${n}_{2}$ are the refractive indices of air and water, respectively^75^) and the tag’s location determined as the point where the rays diverge by 7 mm (i.e., the diameter of the tag in world coordinates)^76^ (Fig. 1a). Tag coordinates are reported as $(x,y,z)$ in mm, where $z$ represents depth from the water surface ($z=0$ mm) to the tank floor ($z=80$ mm); the refractive index of water was assumed to be 1.333^77^.

The accuracy of this approach was assessed by imaging tags placed at 32 known locations distributed approximately uniformly around the $xy$ plane of the tank at 3 different depths (0 mm, 40 mm, and 80 mm), and estimating their position as described above. The overall root-mean-square error (RMSE; the square root of the mean squared distance between the actual and estimated locations) was 1.49 mm (0.92 mm at the surface, 1.25 mm at a depth of 40 mm, and 1.80 mm at the tank floor) (Supplementary Figure S1).

### Characterising individual movement

Statistics summarising the movement of each individual fish over the 60 min (36,000 frames) of data collection were calculated as described below. All data processing and statistical analysis was conducted in R 3.6.3^78^. Missing position data (which occurred in approximately 1.9% of frames, for example when a tag appeared blurred or was obscured by the fish’s body position) was imputed using cubic spline interpolation (with the *na_interpolation* function in the *imputeTS* package^79^) and then noise was removed using a zero-phase forward and reverse digital filter, using the coefficients of a third-order Butterworth filter with the lowest filter cut-off frequency set such that none of the underlying signal was lost^80^. The direction of movement was inferred from the change in the fish’s noise-corrected position between consecutive time steps. In line with previous studies^57,81,82^ we considered the geometric characteristics of the observed movement in terms of step length (the Euclidean distance between two consecutive positions, in mm) and relative turning vector (the relative change in direction between two consecutive positions)^81^.

As has been found in other species^81^, distributions of step lengths could be well approximated by gamma distributions (fitted using the *fitdist* function in the *fitdistrplus* package^83^); see Results. Fits to other putative distributions (including normal, Cauchy, Weibull, logistic, and log-normal distributions) were compared using the Akaike Information Criteria (AIC), but in all cases the difference in AIC scores between these and the best-fitting (gamma) distribution was > 10 suggesting they fitted the data substantially less well^84^. Because step lengths between consecutive time points are unlikely to be independent (i.e., individuals will tend to continue moving at approximately the same speed between successive time points), for each individual we also calculated the first-order autocorrelation in step length at a lag of 0.1 s^85^ using Spearman rank correlations, and tested whether these differed significantly from zero (i.e., independence) using randomisation tests^86^.

Relative turn vectors, representing displacements on the sphere, were modelled using Kent distributions^87^. The Kent distribution can be considered a generalisation of the von Mises–Fisher distribution which allows for distributions of any elliptical shape, size, and orientation on the surface of the sphere^87,88^. They were therefore appropriate for the turn data obtained here which tended to exhibit a greater spread laterally than dorsoventrally (see Results). The probability density function of the Kent distribution is given by

2 $$f\left(\mathbf{x};{\varvec{\theta}}\right)={c\left(\kappa ,\beta \right)}^{-1}\mathrm{exp}\left\{\kappa {{\varvec{\upgamma}}}_{1}^{\mathrm{T}}\mathbf{x}+\beta \left[{\left({{\varvec{\upgamma}}}_{2}^{\mathrm{T}}\mathbf{x}\right)}^{2}-{\left({{\varvec{\upgamma}}}_{3}^{\mathrm{T}}\mathbf{x}\right)}^{2}\right]\right\},$$

where $\kappa$ ($\kappa >0$) determines the concentration or spread of the distribution, $\beta$ ($0\le 2\beta \le \kappa$) determines its ellipticity, $c\left(\kappa ,\beta \right)$ is a normalisation constant, and ${{\varvec{\upgamma}}}_{1},{{\varvec{\upgamma}}}_{2},{{\varvec{\upgamma}}}_{3}$ are orthogonal unit vectors describing the mean, major, and minor axes, respectively^87^. The larger the values of $\kappa$ and $\beta$, the more concentrated and elliptical the distribution will be, respectively. Kent distributions were fitted using the *kent.mle* function in the *Directional* package^89^.

Fitting a Kent distribution to the relative turn vectors for an entire movement path for a given fish tended to result in a very poor fit. The primary reason for this was that the shape of the relative turn vector distribution varied as a function of step length: for example, when the fish were moving fast (i.e., had long step lengths), the distributions tended to be narrow with a pronounced peak in the direction of travel meaning that fast-moving fish were less likely to make abrupt lateral turns; in contrast, when fish were moving slowly (i.e., had relatively short step lengths), the distributions tended to be much broader meaning that slow-moving fish were very likely to abruptly change direction. To accommodate this, the relative turn vector data were subset based on their corresponding step lengths (specifically, we considered subsets spanning 1 mm intervals of step length: [0 mm, 1 mm), [1 mm, 2 mm), and so on, for which the sample size was ≥ 1000), and separate Kent distributions fitted to each subset. The non-linear relationship between step length and the concentration and ellipticity parameters of the associated Kent distribution of relative turn vectors were then modelled using power functions of the general form $g\left(y\right)=k{y}^{d}$, estimated using non-linear least-squares (with the *nls* function in the *stats* package).

To quantify the variation in movement behaviour between individual fish we constructed dissimilarity matrices for both step length and relative turn vectors, using the Hellinger distance^90^ as a measure of dissimilarity between the empirical distributions. These were then visualised using metric multidimensional scaling (MDS) plots by plotting the first two principal coordinates^91^. Finally, we tested whether body size predicted aspects of movement behaviour using distance matrix regressions (implemented using the *MRM* function in the *ecodist* package^92^), in which the response variable was the dissimilarity matrix for either step length or relative turn vectors, and the predictor was a matrix containing the Euclidean distances in standard length between pairs of fish. *P *values were computed using 999 permutations^93^.

### Modelling individual movement

We next constructed a simple model of stickleback movement, parameterised using the empirical data. In the model, individual fish $i$ have a position vector ${\text{c}}_{i}$ and a unit direction vector ${\text{v}}_{i}$ in continuous three-dimensional space. Time is partitioned into discrete time steps $t$ with a regular interval $\tau$, where here $\tau$ is set to 0.1 s to match the sampling rate of the empirical data^94^. At each time step, the position and direction of the fish is updated as follows.

First, a random step length ${s}_{i}(t)$ is drawn from a gamma distribution with shape parameter ${a}_{i}$ and rate parameter ${b}_{i}$. If $t=0$ then this is simply a random draw from this distribution; however, if $t>0$ then a correlated random value is drawn from a bivariate distribution with Gamma(${a}_{i}$,${b}_{i}$) marginals and a given rank correlation structure^95^. Specifically, the Gamma(${a}_{i}$,${b}_{i}$) distributed step length at time step $t-1$ is transformed to a uniform distribution on the unit interval [0,1] by computing its cumulative distribution function (CDF), and then to a standard normal distribution by applying an inverse normal CDF. A normally distributed correlated random value can then be obtained using^96^

3 $${z}_{0}=\rho \cdot {s}_{i}(t-\tau )+\sqrt{1-{\rho }^{2}}\cdot {z}_{1},$$

where ${z}_{1}$ is a random value drawn from a standard normal distribution, and the Spearman’s rank correlation coefficient ${\rho }_{{\text{s}},i}$ (denoting the strength of first order autocorrelation) is mapped to a Pearson’s correlation coefficient ${\rho }_{i}$ using the relationship^97^

4 $${\rho }_{i}=2\mathrm{sin}\left({\rho }_{{\text{s}},i}\frac{\pi }{6}\right).$$

Applying the above steps in reverse transforms ${z}_{0}$ back to a Gamma(${a}_{i}$,${b}_{i}$) distribution, yielding ${s}_{i}(t)$ with the desired rank correlation. This ensures that when ${\rho }_{{\text{s}},i}>0$, consecutive step lengths will show some degree of correlation, allowing fish to exhibit the full extent of movement heterogeneity we observed empirically. This includes, for example, both rapid bursts of movement (when consecutive step lengths are large) as well as sustained bouts of relatively low activity or inactivity (when consecutive step lengths are small or zero).

Next, the direction vector at time $t$, ${\text{v}}_{i}(t),$ is determined by drawing a random unit vector from a Kent distribution, parameterised using the observed power-law relationships between ${s}_{i}(t)$ and both concentration, $\kappa$, and ellipticity, $\beta$ (i.e., as step length increases, the Kent distribution of direction vectors becomes increasingly concentrated and elliptical, albeit non-linearly; see Results). Consistent with the empirical data, the mean direction vector of this distribution is taken to be the heading at the previous time step (so the modal direction of travel is always forwards) and the major and minor axes are assumed to be parallel with the lateral and dorsoventral axes of the fish, respectively. Finally, the fish’s position at time step $t$ is given by

5 $${\text{c}}_{i}\left(t\right)={\text{c}}_{i}\left(t-\tau \right)+{\mathbf{v}}_{i}(t){s}_{i}\left(t\right).$$

### Preference tests

In order to test the ability of the model presented above to realistically simulate stickleback movement behaviour, we ran a Turing-type preference test^29^ in which live fish (*n* = 32) were given a single two-choice association trial where they were presented with two animated conspecifics, each of which moved around the virtual environment using a different set of movement rules. One virtual fish of each pair followed a random movement path simulated using the model, while the other followed a section of real movement path extracted directly from the empirical data. In each case, movement paths were paired so that real and simulated paths had the same movement statistics (i.e., were based on data from the same, randomly selected real individual).

The general construction and presentation of the animated fish closely followed the procedure described in Pike^17^. The stimulus consisted of a digital model of a three-spined stickleback (TurboSquid, Product ID: 172706) that was coloured uniform grey, except for the fins (which were white and semi-transparent) and pupils (which were black). The model was sized so that it appeared life-sized (i.e., 41.8 mm standard length) when at the ‘front’ of its virtual environment. This environment was uniformly grey and contained no landscape features. Animations were generated in real time using custom-written Matlab (MathWorks, Natick, MA) functions^17^, and were presented at 10 frames per second to match the rate at which the empirical data were sampled.

For simulated movement, parameter values were randomly selected from one of 59 measured individuals (one fish was excluded because it exhibited almost no movement throughout the period of data collection; see Results). Movement was simulated within a virtual circular tank with the same dimensions as the test tank. The fish started at a random location (drawn from a random uniform position within the cylindrical volume of the tank), and movement paths were generated over 1200 timesteps (2 min). To constrain movement within the tank’s boundaries, if the fish’s estimated position three timesteps in the future (assuming it stayed on the same trajectory and maintained the same step length) fell outside the tank, random values of step length, ${s}_{i}\left(t\right)$, and direction vector, ${\mathbf{v}}_{i}(t)$, were repeatedly drawn until its estimated position fell within the tank boundary. This ensured consistency in a fish’s movement statistics (Supplementary Figure S2). Real movement paths consisted of 2 min sections of movement extracted from the empirical movement data, using a randomly determined start time.

To confirm that focal fish were using characteristics of the virtual fish’s movement to inform their choice, rather than simply responding to the presence of a conspecific, we also ran a second test in which focal fish (*n* = 32 naïve individuals) were presented with a binary choice between two virtual fish that differed in how ‘natural’ their movement was. In these trials both fish exhibited simulated movement (as described above), except that for one fish of each pair step lengths ${s}_{i}(t)$ were drawn from a two-point distribution on $[0, 20]$, with the probabilities of drawing each value set so that both fish had the same mean step length. Turns were determined in the same way as in the main model. This caused it to move in an unnaturally stilted manner, at least to human observers (Supplementary Figure S2).

For each trial, an experimental fish was selected haphazardly from one of four holding tanks and transferred individually to a transparent plastic cylinder (8 cm diameter) located in the centre of a 33 × 18 × 19 cm glass experimental tank filled with 10 cm water. The long sides of the tank were covered with opaque white card, while calibrated 22 inch flat-screen CRT monitors (Iiyama VisonMaster 513, MA203DT; 800 × 600 px resolution, 180 Hz refresh rate) placed at each end of the tank were used to display the animated stimulus fish^17^ (Supplementary Figure S3). The fish was immediately shown two simultaneous animation sequences, one presented on each monitor. Which monitor (i.e., which side of the tank) showed the simulated movement was randomised for each trial. After 30 s the focal fish was released from the cylinder and was able to swim freely within the experimental tank for a further 90 s. Throughout each trial the experimental tank was monitored by a camera mounted directly overhead, allowing the position of the focal fish to be tracked in real time^17^. For data collection, the tank was divided into three virtual zones, one immediately in front of each monitor (with a width of 5 cm, termed ‘choice zones’) and one in the centre of the tank (‘neutral zone’); if the fish entered one of the choice zones (which we defined as any part of the focal fish’s body crossing the zone demarcation line) it was assumed to be exhibiting a choice. We recorded which choice zone was entered first and the latency (s) to make this choice. After the trial ended the fish was removed from the test tank and replaced in a different holding aquarium to ensure that no fish was used more than once.

We assessed focal fishes’ relative preference for the virtual fish of each pair that followed a movement path simulated by the model, over the one following either a real or an ‘unnatural’ movement path, by comparing first-choice preferences using binomial tests. We also tested whether the latency to make their first choice differed between stimuli using two-sample t-tests. However, if focal fish were unable (or unwilling) to differentiate between the different types of movement path, then we would fail to reject our null hypothesis of no deviation from chance levels of preference. It was therefore important that our sample afforded sufficient statistical power to minimise the possibility that any null result is in fact a false negative (Type II error). We determined the minimum sample size necessary to detect a ‘medium’ effect (sensu Cohen, 1988) of first-choice preference with a power of 80% using the *pwr.p.test* function in the *pwr* package for R^98^, yielding n = 32.

### Supplementary Information

Supplementary Figures.

## Data Availability

The datasets generated and analysed during the current study are available in the University of Lincoln Repository, https://doi.org/10.24385/55426. Code is available on GitHub, https://github.com/thomaswpike/fishsim.
