# Supplementary material for: Simulating individual movement in fish
Source: Sci Rep. 2023 Sep 4;13:14581. doi: 10.1038/s41598-023-40420-1 (PMC10477313; doi:10.1038/s41598-023-40420-1)
Supplement: Supplementary file 1 — Supplementary Figures. [file 41598_2023_40420_MOESM1_ESM.docx]

**Supplementary Information**


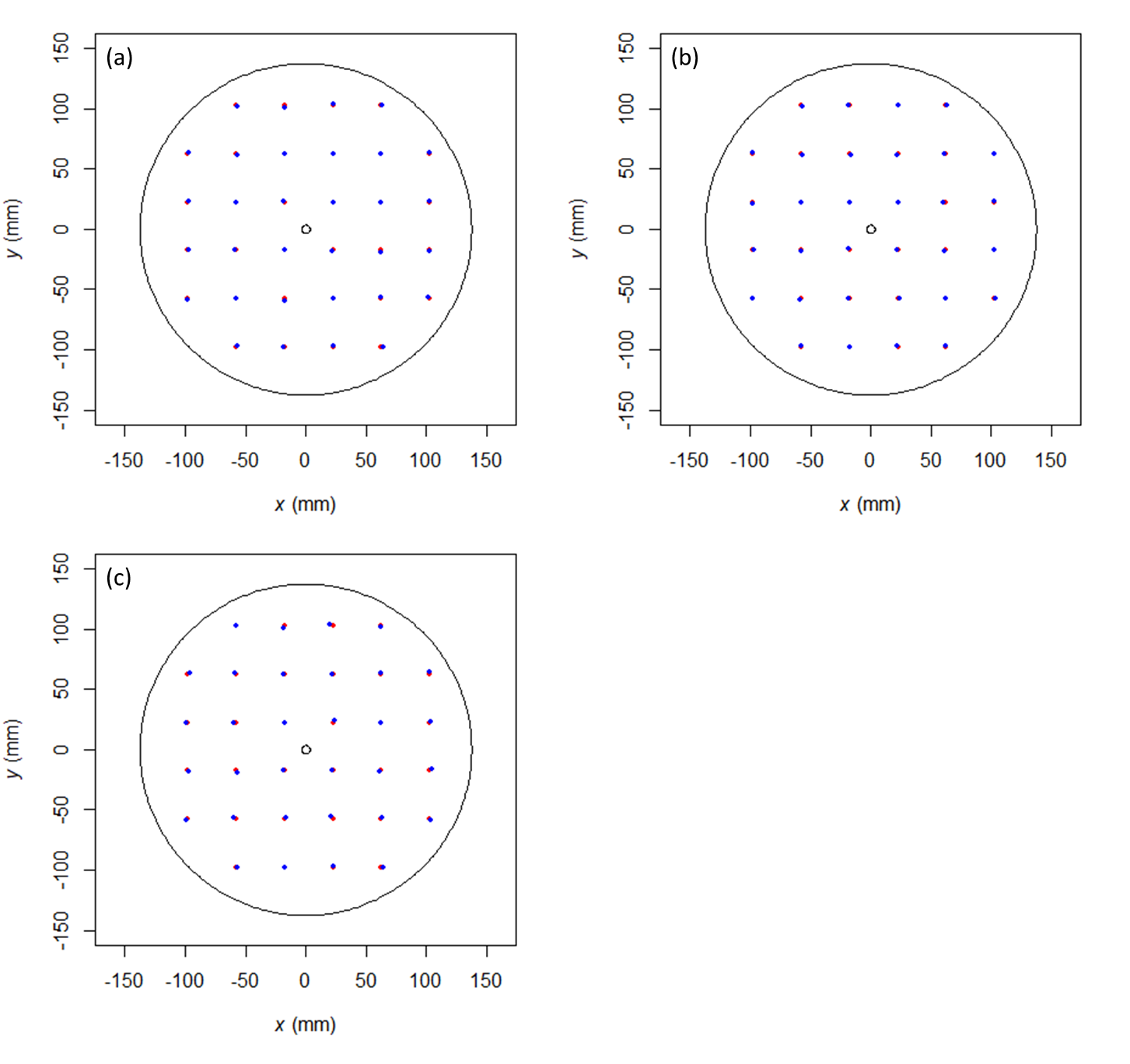


Figure S1. Accuracy with which the position of the tag could be determined. Red dots denote the known position of tags placed approximately uniformly at 32 locations around the $xy$ plane of the tank at 3 different depths, (a) 0 mm (i.e., at the water surface), (b) 40 mm, and (c) 80 mm (i.e., at the bottom of the tank), and blue dots represent the positions estimated using the procedure described in this paper.


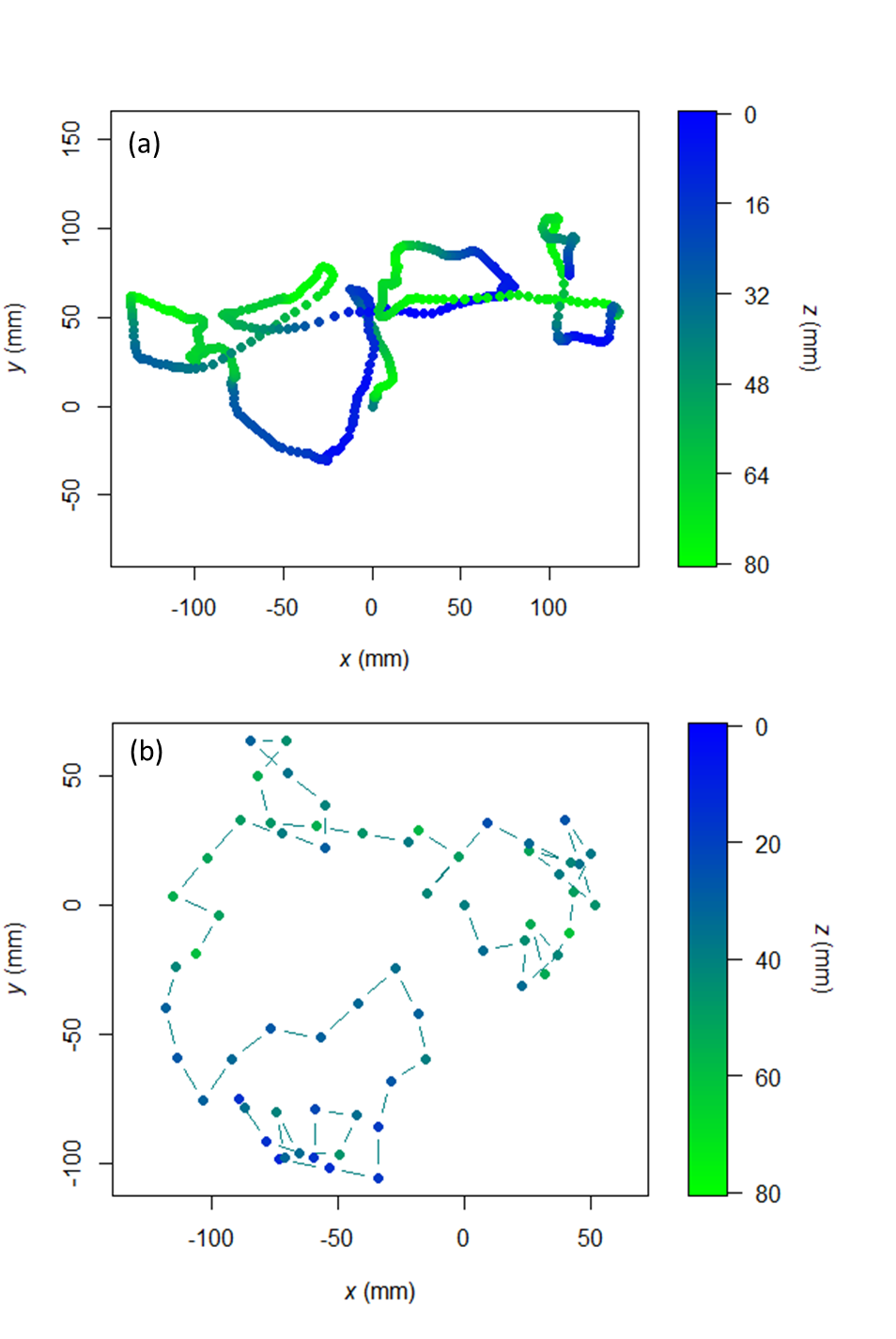


Figure S2. Representative 10 s movement paths (a) simulated using the model described in this paper and (b) showing simulated ‘unnatural’ movement. See text for full details. Each point denotes the fish’s position at 0.1 s intervals, with the colour representing depth ($z = 0$ is at the water’s surface). Note that in (b) often many of the points overlap (i.e., the fish remained in exactly the same position for several time steps before moving), with lines indicating the locations of consecutive positions.


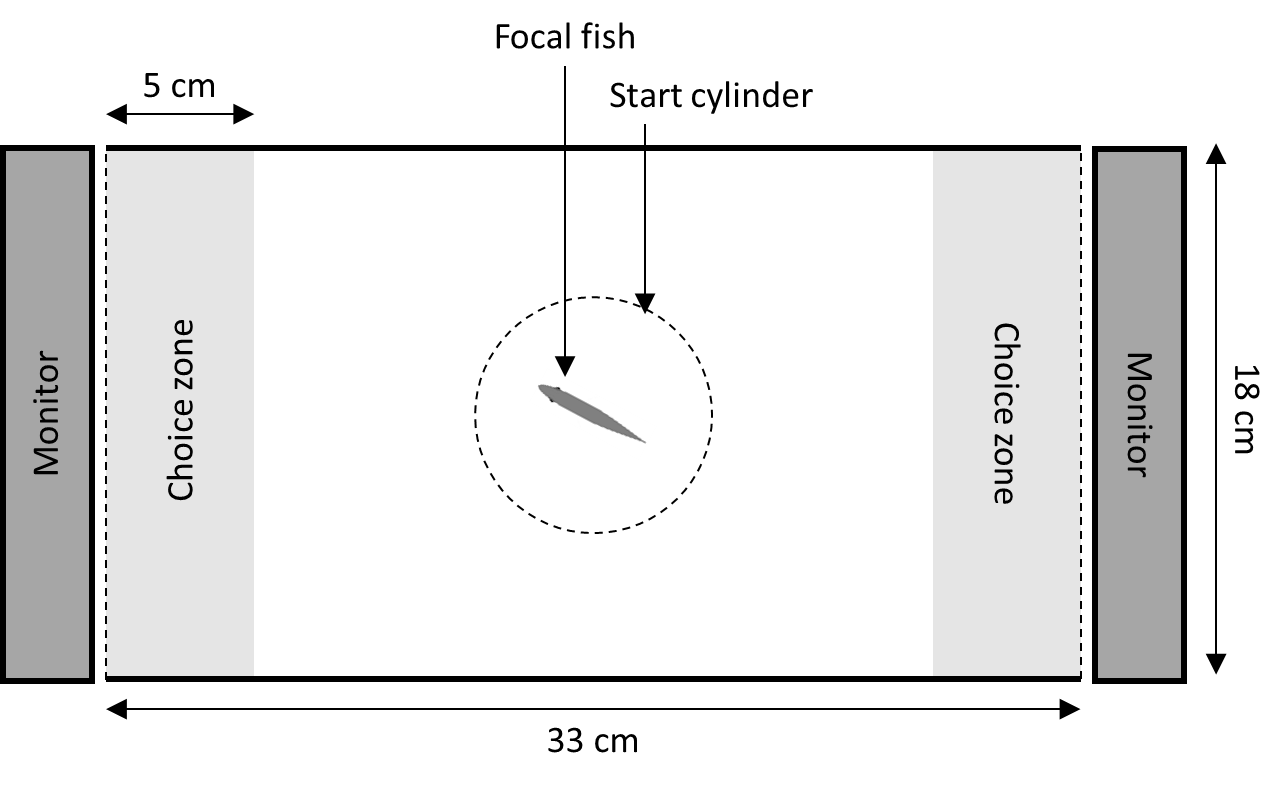


Figure S3. The experimental setup used for the preference tests, in which the tank is drawn to scale. Dashed lines denote a transparent wall, while thick solid lines indicate an opaque barrier; choice zones were not physically demarked and are shown for illustration only. The water level of the tank was 10 cm.
